# The impact of standardizing the definition of visits on the consistency of multi-database observational health research

**DOI:** 10.1186/s12874-015-0001-6

**Published:** 2015-03-08

**Authors:** Erica A Voss, Qianli Ma, Patrick B Ryan

**Affiliations:** Janssen Research & Development, 920 Route 202, Raritan, NJ 08869 USA

**Keywords:** Administrative claim database, Inpatient visit, Standardization

## Abstract

**Background:**

Use of administrative claims from multiple sources for research purposes is challenged by the lack of consistency in the structure of the underlying data and definition of data across claims data providers. This paper evaluates the impact of applying a standardized revenue code-based logic for defining inpatient encounters across two different claims databases.

**Methods:**

We selected members who had complete enrollment in 2012 from the Truven MarketScan Commercial Claims and Encounters (CCAE) and the Optum Clinformatics (Optum) databases. The overall prevalence of inpatient conditions in the raw data was compared to that in the common data model (CDM) with the standardized visit definition applied.

**Results:**

In CCAE, 87.18% of claims from 2012 that were classified as part of inpatient visits in the raw data were also classified as part of inpatient visits after the data were standardized to CDM, and this overlap was consistent from 2006 to 2011. In contrast, Optum had 83.18% concordance in classification of 2012 claims from inpatient encounters before and after standardization, but the consistency varied over time. The re-classification of inpatient encounters substantially impacted the observed prevalence of medical conditions occurring in the inpatient setting and the consistency in prevalence estimates between the databases. On average, before standardization, each condition in Optum was 12% more prevalent than that same condition in CCAE; after standardization, the prevalence of conditions had a mean difference of only 1% between databases. Amongst 7,039 conditions reviewed, the difference in the prevalence of 67% of conditions in these two databases was reduced after standardization.

**Conclusions:**

In an effort to improve consistency in research results across database one should review sources of database heterogeneity, such as the way data holders process raw claims data. Our study showed that applying the Observational Medical Outcomes Partnership (OMOP) CDM with a standardized approach for defining inpatient visits during the extract, transfer, and load process can decrease the heterogeneity observed in disease prevalence estimates across two different claims data sources.

**Electronic supplementary material:**

The online version of this article (doi:10.1186/s12874-015-0001-6) contains supplementary material, which is available to authorized users.

## Background

Managed care administrative claims databases are widely used in many pharmacoepidemiology studies [1]. The medical claims data are derived during the reimbursement process, and are generated from two standard forms: Health Insurance Claim Form (HCFA-1500) and Universal Billing form (UB-92). Health care services provided by a single practitioner or practitioner groups are submitted for payments on the HCFA-1500 form, while hospital inpatient and acute care outpatient services are submitted for payments on the UB-92 format. While a secondary use of the information, these claims databases are of great benefit for observational research as they provide information on large, heterogeneous populations of insured patients that are geographically dispersed at generally lower costs than those would be gathered by prospective data collection and randomized clinical trials [1,2].

One challenge that researchers face using administrative claims databases from multiple sources is the lack of consistency in the structure of the underlying data. Claims data are maintained and used for research through various institutions, including government agencies (e.g. Centers for Medicare and Medicaid Services Research Data Assistance Center [CMS ResDAC]), large payers with affiliated research arms (e.g. HealthCore, Optum), or claims processors who aggregate and license data (e.g. IMS, Truven). Each of these organizations may store claims data in different technical environments (e.g. SAS, Microsoft SQL Server, Oracle) and have developed their own internal data structures to record the claims information. The design of the data structure can influence how the specific data elements within source claims from the HCFA-1500 and UB-92 forms are represented and organized. For example, in the Truven claims database [3], medical claims are partitioned into separate tables for inpatient and outpatient services with the inpatient services claims being classified by the presence of a ‘room and board’ revenue code. In the Optum Clinformatics database [4], all medical service claims are maintained in a single data table which contains a field to indicate claims associated with an inpatient confinement. In both cases, the choice of data structure and definition of inpatient classification could be considered reasonable approaches in preparing the data for research purposes. However, the assumptions that underlie the source structure are rarely described in any detail within published research on these databases. It is unknown whether the inconsistency in inpatient definition when taken across data vendors can have a material impact on research findings or negatively impact the ability to conduct cross-database comparisons of analysis results.

Due to these variations in format and variable definition, different researchers might make different decisions on how to process the data as part of analysis and these decisions can affect the results [5]. Even a simple choice of using primary diagnosis versus any diagnoses of a condition within the same administrative claim database can make a difference in the cohort being studied. For example, it has been found that the use of a primary diagnosis of pneumonia and influenza discharges to estimate influenza-associated hospitalization will not fully capture the influenza cohort [6]. Being able to correctly define health care utilizations (i.e. visit types) is another example of an important decision that researchers need to make. When using administrative claim databases for observational studies, many researchers use the type of visits as a proxy for severity of disease and it is often believed among some practitioners that inpatient diagnoses are more reliable than outpatient diagnoses for some conditions. For example, the Mini-Sentinel pilot project conducted literature reviews on the accuracy of administrative claims diagnostic codes and found that when defining seizures, convulsions, or epilepsy from electronic healthcare data, positive predictive value ranged on the visit types: >90% for emergency room (ER) visits, 59.7-79.1% for inpatient, and extremely low for outpatient [7]. Since the definition of visit types can vary among different claim databases, the lack of standardization can serve as an additional source of systematic error in any analysis where the type of visits is important to the study design.

Currently there are many data network efforts trying to reduce the variations among different databases by converting different observational healthcare databases into a standardized format (i.e. common data model [CDM]), such as Mini-Sentinel [8], the Observational Medical Outcomes Partnership (OMOP) [9,10], EU-ADR [11], the National Patient-Centered Clinical Research Network (PCORnet) [12], or Health Maintenance Organization (HMO) Research Network (HMORN) [13]. By converting data into the same format, one objective of those efforts is to reduce the systematic error caused by variations in format among different databases. Standardization should increase transparency and improve the ability of networks to replicate study results across multiple institutions. A common challenge experienced across all of these efforts is that standardizing a data model structure - and in some instances, the content through standardized vocabulary - does not ensure standard conventions for handling specific information (visit types, reversals etc.) are consistently applied across participating institutions. Since the administrative claim databases in the U.S. are derived from standard reimbursement information, we believe it is possible to develop standard definitions when such databases are converted into CDMs even if they vary in format or have different source representations.

This paper describes the development of a standardized definition for an inpatient visit in administrative claim databases using revenue codes. Since the data is originally derived from standardized forms, converting it into standard format may offer a great opportunity to improve consistency of the underlying data used for multi-database research. We believe revenue and place of service codes should provide an avenue to such standardization since they are primarily used for reimbursement purposes and always available in administrative claim databases. We applied such a common definition to two different claims databases (Truven MarketScan Commercial Claims and Encounters [CCAE] and Optum Clinformatics [Optum]) while converting the raw data into a CDM and evaluated whether this transformation and standardization improves consistency in analysis results between the two databases. While there is no gold standard source of truth on a patient’s visit status, we believe that applying a consistent approach to its definition can improve transparency and reproducibility in observational research. Our main motivation for this study was to assess the impact of applying a standard visit definition to two claims databases on the prevalence of health service utilization and disease prevalence observed during inpatient encounters.

## Methods

### Data sources

The CCAE and Optum databases were used in this study. These databases were chosen for study because they are two large claims databases with have similar distributions in types of private insurance (e.g., preferred provider organization [PPO], HMO), age, and gender. The CCAE database primarily consists of privately insured population and captures administrative claims with patient-level de-identified data from inpatient and outpatient visits and pharmacy claims of large employers and multiple insurance plans as well as patient’s enrollment information (e.g., demographics, period of enrollment, plan type). The version of CCAE database used for this analysis contained over 140.6 m lives with medical and/or pharmacy coverage, with data from 1/1/2000 to 10/31/2013. Optum contained 37 m privately insured lives with data from 10/01/2005 to 12/31/2012 and all its lives have both medical and pharmacy coverage. The two primary differences between Optum and CCAE are that 1) CCAE aggregates data from multiple payers while Optum is primarily representative of one large payer and 2) CCAE contains only privately-insured patients, while Optum contains both privately-insured patients and Medicare beneficiaries. To make the databases more comparable, the Medicare patients in Optum were removed for this analysis. Nothing a priori would suggest that these populations would differ radically in rates of disease by age and gender strata. More detailed information on the tables utilized in the study can be found in Additional file 1.

### Sample selection

To ensure the populations from two databases had similar demographic distributions, we only included members who had 2012 full-year enrollment of commercial based insurances with both medical and pharmacy coverage. We also excluded members whose ages in 2012 were greater than 65 or members with unknown gender. Medical claims of eligible members with service dates within 2012 were used for our analysis. We also applied the same criteria for selecting eligible populations in other years (2006–2011) and repeated those analyses after-mentioned in each of these years.

### Study measures

#### Definition of inpatient visits in raw data

While the source data for Optum and CCAE come from the same forms (UB-92 and HCFA-1500) the data vendors organize the results in different ways. Optum has created inpatient confinements in a medical claims table to capture inpatient episodes occurring in an acute care hospitalization or skilled nursing facility setting. Any Optum records with an associated confinement identifier, an Optum derived field, were considered as part of an inpatient visit unless they were identified as ER claims by the place of service field (this was recommended as an inpatient visit definition from Optum). CCAE has defined inpatient admissions by grouping service records meeting certain criteria (e.g. a room and board claim must be present) into one table; all claim records, except ER claims, in this table were considered as inpatient visits (ER claims in CCAE can be identified by service category). The codes for defining ER claims in raw data can be found as part of Additional file 2.

#### Definition of inpatient visits in CDM

**Figure 1:**
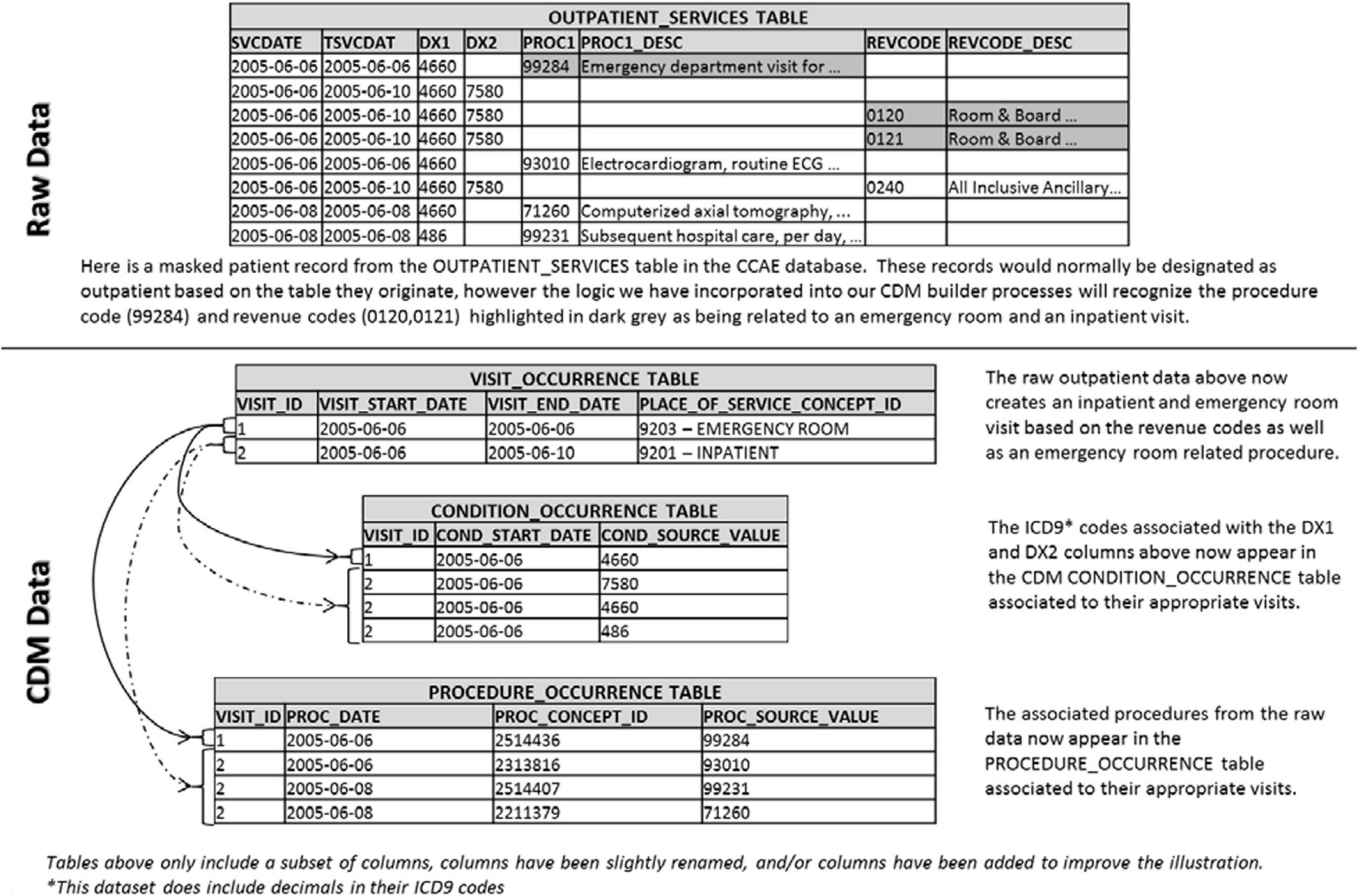
**Truven MarketScan Commercial Claims and Encounters (CCAE) reclassification of a single patient’s outpatient record to inpatient or emergency room visit.**

The raw data was converted into the OMOP CDM Version 4 format [10,14]. Detailed documentation for the conversion of these databases into the CDM format can be found on the OMOP website [9]. When transforming the raw data into the OMOP CDM format, we applied a standardized classification approach to define inpatient visits through the extract, transform, and load (ETL) processing. We first assigned a claim type for each medical claim record: If a record contained any of the revenue codes for inpatient visits [15], it was assigned as an inpatient claim; else if a record contained a place of service code, revenue code, procedure code, or service sub-category code (CCAE only) for an ER claim, it was assigned as ER claim; other records were assigned as an outpatient claim. Then for each patient, we collapsed records of inpatient claims together as long as the time between the service end date of one record and the service start date of the next was less than or equal to one day apart, and each consolidated record was considered as one inpatient visit. If an ER or outpatient claim occurred during an inpatient visit, it would have been consolidated into the inpatient visit unless it was an ER claim that occurred on the first day of the inpatient visit. The codes for defining inpatient or ER claims in both Optum and CCAE can be found in Additional file 2. Figure 1 provides an example of a claim from CCAE’s outpatient services table and how it was re-categorized to an inpatient visit.

### Analysis

We estimated the prevalence of all inpatient conditions to evaluate the impact of our standardized approach. Inpatient conditions were defined based on diagnosis codes observed in the primary position or any of the secondary positions within the medical claims. Diagnosis codes in both databases were natively coded in International Classification of Diseases, Ninth Revision (ICD-9), and all ICD-9 codes were consistently grouped into Systematized Nomenclature of Medicine – Clinical Terms (SNOMED-CT) using the ICD-9 – SNOMED mapping made available within the OMOP vocabulary [16]. For each database, the prevalence of each condition in inpatient visits in raw data was compared to that defined in the CDM. We also compared the prevalence of each inpatient condition in the Optum raw data and the CDM to that in the CCAE raw data and the CDM, respectively. The standardized difference (SD) was also used to compare the differences of prevalences of the top ten SNOMED-CT conditions [17]. We evaluated concordance between the raw and CDM through R^2^ correlation statistic. We fit four linear regression models to the prevalence estimates of all conditions: comparing the raw data versus the CDM for both CCAE and OPTUM and additionally comparing the prevalence between Optum and CCAE for the CDM and RAW. Data preparation and analysis were performed using SAS® version 9.3_M1, SAS Institute Inc. Spotfire® 5.5.0, TIBCO® Software Inc. was used to generate visualizations and display the y-intercept (a), slope (b), and R^2^ of the regression (r2).

### Ethics

The analyses using the Optum and CCAE databases were reviewed by the New England Institutional Review Board (NEIRB) and determined to be exempt from board IRB approval as this research project involved no risk to the subjects and does not meet the definition of human subject research (NEIRB#12-284 & NEIRB# 12–286). We obtain these de-identified patient-level dataset through a license agreement with each data holder.

## Results

**Figure 2:**
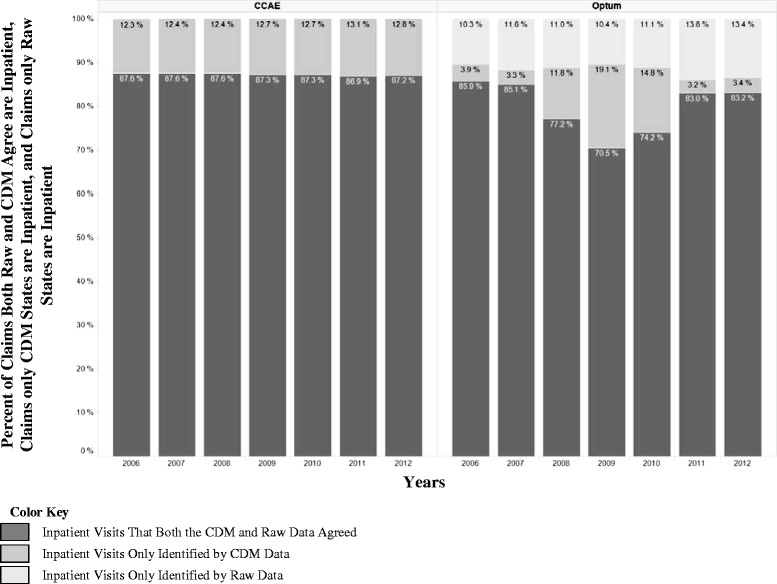
**Inpatient claims reclassification between raw and CDM data for 2006 through 2012.**

**Figure 3:**
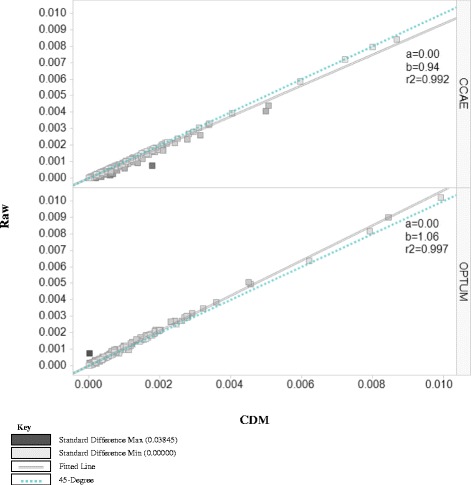
**2012 prevalence of SNOMED-CT conditions in raw versus CDM in optum & CCAE.**

**Figure 4:**
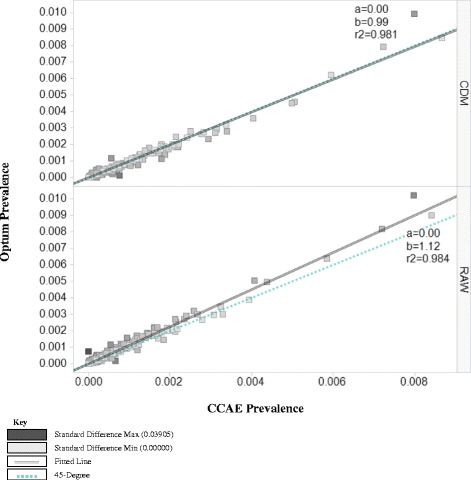
**2012 prevalence of SNOMED-CT conditions for CCAE versus optum in the raw & CDM datasets.**

Table 1 reports the demographic distribution of eligible members in CCAE and Optum during the year of 2012. CCAE has more than 3 times as many active members in 2012 as Optum. The two databases are similar in terms of age and gender. Our stratified analysis by age and gender showed such demographic differences had no impact on the findings in Figures 2, 3 and 4. The demographic profile in CCAE for 2012 was similar to the demographic profile for Optum during the same period. The demographics in both databases were consistent from 2006 through 2011.

**Table 1:** **Demographics during 2012**

**Statistic**	**CCAE**	**Optum**
**Number of eligible members**	28,747,530	7,924,173
**Age, Mean (SD)**	34.67 (18.35)	34.29 (18.04)
**Age Decile, %**		
**00-09**	11.21	11.48
**10-19**	15.53	14.79
**20-29**	13.57	13.77
**30-39**	14.33	16.02
**40-49**	17.68	18.46
**50-59**	19.44	18.03
**60-65**	8.24	7.45
**Gender, %**		
**Female**	51.35	50.27

SD = Standard deviation.

We illustrate the reclassification of claims which were classified as inpatient claims in raw data or the CDM for each year from 2006 to 2012 in Figure 2. In 2012, among claims that were classified as inpatient visits by the raw data or the CDM, 87.18% were in the overlap and 12.82% were only in the CDM (i.e. defined as outpatient claims in the raw data) for CCAE while for Optum those numbers were 83.18% and 3.43% respectively. We also found that CCAE followed the same inpatient classification pattern exhibited in 2012 among years from 2006 to 2011, while Optum varied in this classification (notably in 2008, 2009, and 2010 the percent of records classified as inpatient only in the CDM were 11.77%, 19.12%, and 14.75% respectively instead of around 3-4% as we see in other years).

We then examined the impact of the visit classification on the prevalence of diseases in an inpatient setting. Table 2 shows the top ten selected SNOMED-CT conditions, and the prevalence estimates in the raw data and the CDM. In most situations, we found the standardized difference of the prevalence of a specific condition between two databases was less in the CDM than in the raw data. For example, we see the prevalence of the concept of “Abdominal Pain” in CCAE goes up from 0.41% in the raw data to 0.50% within the CDM and the prevalence in the Optum data goes down from 0.50% in the raw data to 0.45% within the CDM, and the two databases have more consistent prevalence estimates after standardization to CDM. In total, amongst 7,039 conditions reviewed, the difference in the prevalence of 67% of conditions in these two databases was reduced after standardization, while 23% of condition prevalence estimates did not change after standardization. In the remaining 10% of conditions, the prevalence between the two databases became less consistent after standardization. To review the changes for all the conditions reviewed, please see the Additional file 3.

**Table 2:** **Top 10 conditions of SNOMED-CT inpatient conditions in the raw versus the CDM data format**

	**Raw data**			**CDM data**		
**SNOMED-CT concept**	**CCAE PR (%)**	**Optum PR (%)**	**SD**	**CCAE PR (%)**	**Optum PR (%)**	**SD**
Essential hypertension	0.84	0.90	0.006	0.87	0.84	0.002
Single live birth	0.80	1.02	0.024	0.80	0.99	0.020
Mother delivered	0.72	0.82	0.011	0.72	0.79	0.008
FTND	0.58	0.64	0.007	0.60	0.62	0.003
Chest pain	0.44	0.50	0.008	0.51	0.46	0.007
Abdominal pain	0.41	0.50	0.015	0.50	0.45	0.007
Hyperlipidemia	0.39	0.39	0.001	0.40	0.36	0.007
GERD	0.33	0.30	0.005	0.34	0.28	0.011
T2DM	0.32	0.35	0.004	0.34	0.32	0.003
Dyspnea	0.26	0.32	0.011	0.31	0.29	0.004

CCAE = Truven MarketScan Commercial Claims and Encounters; Optum = Optum Clinformatics; CDM = Observational Medical Outcomes Partnership (OMOP) Common Data Model; FTND = Full term normal delivery; GERD = Gastro-esophageal reflux disease; T2DM = Type 2 diabetes mellitus; PR = Prevalence; SD = Standardized Difference.

Figure 3 shows the comparison of prevalence of all SNOMED-CT inpatient conditions in the raw data to that in the CDM for both databases. Two lines within the graphic are used to visualize the changes in prevalence of conditions in the databases: the dotted blue line represents a 45-degree and the double black line is a fitted line. If the raw and the CDM data were identical, the prevalence of all inpatient conditions would fall on the 45-degree line. However, we observed the prevalence of inpatient conditions in CCAE raw was lower than the prevalence estimates from within the CDM (beta coefficient [b] = 0.94) while in Optum inpatient condition prevalences were lower in the CDM than the raw data (b = 1.06). The increase with CCAE was expected based on our how standardization method was applied to that database, but the directionality of the shift in prevalence within of Optum was not prescribed by the algorithm. This confirms the finding in Figure 2 that CCAE raw data defined less inpatient claims while Optum raw data defined more inpatient claims than the standardized approach. We performed the same analysis for the previous years, and the results confirmed the finding in 2012.

In Figure 4, we compared the prevalence of 2012 inpatient conditions in Optum raw data and CDM to that in CCAE raw data and the CDM, respectively. Before standardization, we found that on average each condition in Optum was 12% more prevalent than that same condition in CCAE (b = 1.12); after standardization, there was only a 1% mean difference in the prevalence estimates between the two databases (b = 0.99).

## Discussion

One of the challenges of using administrative claims databases is database heterogeneity [18]. We typically consider heterogeneity to arise from different studies using data sampled from different source populations using different data capture processes. One source of heterogeneity that may be less commonly considered among administrative claims databases may be the way data holders process the raw data from the HCFA-1500 and UB-92 forms. Although there are many efforts trying to minimize the impact of the variations among claim databases from different data holders by applying a common data model, bias can still be introduced since different data holders may choose different rules to convert their databases into a CDM. One specific occasion involves defining inpatient visits. Here we evaluated the application of a standard classification of inpatient admissions to two administrative claims databases when converting them into the OMOP CDM and reviewed how that changed consistency between the datasets.

Our study showed that there are differences between the databases in defining inpatient visits in the raw data. As illustrated in Figure 3, CCAE seems to have less inpatient admissions in the raw data, while Optum had more. If we were to accept the raw data’s definitions for inpatient, there is the potential for that to slightly skew the data one way or another depending on the source that you are using. We also found in Figure 2 that within one raw data source you may have variation year to year in how claims visits are being categorized.

However, when we converted the two databases into the CDM and applied the standardized approach for defining inpatient visits during ETL process, the two databases became more comparable. As illustrated in Figure 4, the prevalence of inpatient conditions became similar between the two databases in the CDM. Our approach was revenue codes based and thus independent of how each source handles the data derived from HCFA-1500 and UB-92 forms. Each approach taken by the data holders is not inherently incorrect, but the lack of consistency across sources presents an additional source of systematic error that could be introduced into the analysis process when comparing results across databases. For example, CCAE mentions in their documentation that a small percentage of inpatient services fall into their outpatient services table when no charges are found, just a choice made for their processing however this could be inconsistent with other data holders [19]. Therefore by leveraging the process of the ETL we can create consistency with the definition of inpatient visits across multiple claims datasets. Our ETL conversions of the raw data have applied a consistent algorithm based on common elements in each source database. We did not evaluate the impact of alternative algorithms that could have been considered. Further research would be required to determine if a specific algorithm had better operating characteristics than alternatives.

This study demonstrates the impact of standardization on encounter classification and disease prevalence. It is important to reinforce that the motivation for this work was to improve consistency in research results across databases by applying a common logical approach and set of assumptions between sources. One limitation of this analysis is the lack of available reference standard to compare the prevalence estimates. The two populations are different and therefore we do not know the extent to which we could expect inconsistency. We have made the populations comparable through stratifications by age, gender, and year, but it may be underlying disease and health service utilization patterns account for differences. For these reasons, it is important to focus on the relative comparison from the raw data to CDM-transformed data, rather than the absolute prevalence when evaluating the impact of applying a consistent algorithm across sources. In addition, we were not able to conduct source record verification to assess the validity of the assumptions or to gauge whether one data source’s original data structure is more or less reliable than another in their ability to properly classify inpatient encounters. It would be desirable to develop a common approach that can be demonstrated to improve the reliability of the information, but even in the absence of this evidence, we believe establishing a common approach that can be applied uniformly across all databases has tremendous value.

This analysis provides a descriptive characterization of the impact of standardization on the prevalence of impatient visits and associated diseases. We did not provide formal statistical test or compute measures for inter-rater agreement, but instead relied on the distribution of prevalence estimates and regression coefficients as the means of illustrating the variability observed between databases, before and after standardization. Further analysis could be performed to test the impact, and those approaches could be applied across other databases to further assess the generalizability of these findings. Also, further work should be considered to assess how standardization may impact length-of-stay and cost summarization commonly used in health economic analyses.

Standardizing definitions for visits is just one example of an opportunity for improving consistency in observational research. The use of common data models in efforts such as OHDSI, Mini-Sentinel, and PCORNet present opportunities to establish shared conventions that go beyond the basic data structure and content to impart consistent interpretation of each data elements within a source. Work to standardize analytical methods, as has been done by OMOP and Cancer Intervention and Surveillance Modeling Network (CISNET), is another important step in this direction. The promise of standardizing the entire analysis process, from data management through analysis execution and results interpretation, should offer the ability to simultaneously improve the efficiency, reliability, and reproducibility of research activities.

When it comes to cross-database research, we support the adage that it is better to be consistently wrong than inconsistently right. That is, a primary challenge to research across networks of disparate databases is managing the various sources of error that can exist within each source and determining how to reconcile those errors when combining results across sources. Establishing common standards and assumptions for data process will reduce the variance in systematic error across the network, even if some error based on the common standard persists. To support the community in applying consistent assumptions, we have made the ETL processes we developed publically available [20,21], however this code would still need to be configured to work within each data network and may not be directly applicable to other structures used to store claims data.

## Conclusions

Observational data networks are becoming an increasingly important mechanism for real-world evidence generation, and the success of the networks will largely be determined by their ability to generate consistent analysis results across each participating data source within the network. Many observational data networks have adopted a common data model as a tool to promote greater consistency across the participants. The ETL process of the source data into the CDM provides an explicit opportunity to impose common standards across the data networks. Our study showed that applying the OMOP CDM with a standardized approach for defining inpatient visits during the ETL process can decrease the heterogeneity observed in disease prevalence estimates across two different claims data sources. For 90% of conditions, standardization improved or maintained the consistency of prevalence estimates, with an average prevalence difference between the two databases narrowing from 12% to 1% after standardization. It is important to note that for 10% of conditions, the prevalence estimates after standardization were less consistent; this finding underscores the need for the research community to gain a complete understanding and provide full transparency to all data transformation activities that occur from the source through analyses, as these manipulations can materially impact study results. Defining visits is only one of the many conventions that are needed to enforce consistency across a data network; further community efforts are required to establish consistent approaches for data quality assessment for other data domains. The application of these common standards across databases is a necessary, but not sufficient, step to improving the consistency, transparency, and reliability of observational research.

## Additional files

Additional file 1:
**Specific Details on Table Layouts in Raw Data.** CCAE = Truven MarketScan Commercial Claims and Encounters; Optum = Optum Clinformatics. Additional file 2:
**Codes for Defining Inpatient and Emergency Room Claims Used to Define Standardize Visits.** CCAE = Truven MarketScan Commercial Claims and Encounters; Optum = Optum Clinformatics; CPT = Current Procedural Terminology Codes. Additional file 3:
**Comparing Prevalences and Standardized Differences between the Raw and CDM data in both Optum and Truven.**

## Abbreviations

CCAE: Truven MarketScan Commercial Claims and Encounters
Optum: Optum Clinformatics
CDM: Common data model
OMOP: Observational Medical Outcomes Partnership
HCFA-1500: Health insurance claim form
UB-92: Universal billing form
CMS ResDAC: Centers for Medicare and Medicaid services research data assistance center
ER: Emergency room
PCORnet: National Patient-Centered Clinical Research Network
HMO: Health maintenance organization
HMORN: HMO Research Network
PPO: Preferred provider organization
IRB: Institutional review board
ETL: Extract, transform, and load
ICD-9: International Classification of Diseases, Ninth Revision
SNOMED-CT: Systematized Nomenclature of Medicine – Clinical Terms
SD: Standardized difference
a: y-intercept
b: Slope
r2: R-squared of the regression
R&B: Room and board
FTND: Full term normal delivery
GERD: Gastro-esophageal reflux disease
T2DM: Type 2 diabetes mellitus
CPT: Current procedural terminology codes
