# Supplementary material for: The impact of standardizing the definition of visits on the consistency of multi-database observational health research
Source: BMC Med Res Methodol. 2015 Mar 8;15:13. doi: 10.1186/s12874-015-0001-6 (PMC4369827; doi:10.1186/s12874-015-0001-6)
Supplement: Additional file 1: — Specific Details on Table Layouts in Raw Data. CCAE = Truven MarketScan Commercial Claims and Encounters; Optum = Optum Clinformatics. [file 12874_2015_1_MOESM1_ESM.docx]

**Additional file 1 Specific Details on Table Layouts in Raw Data**

|  | |
| --- | --- |
| **Database** | **Tables** |
| CCAE | ENROLLMENT_DETAIL – Contains the enrollment information of each member, including gender, birth year, enrollment start and end dates, plan type.  DRUG_CLAIMS – Contains prescription drug claims.  INPATIENT_ADMISSIONS – Contains records that summarize information about a hospital admission. It was created by grouping service records meeting certain criteria (e.g., a room and board claim must be present).  INPATIENT_SERVICES – Contains all claim records associated with an inpatient admission.  OUTPATIENT_SERVICES – Contains all other claim records that cannot be associated with inpatient admissions.  FACILITY_HEADER – Contains complete header information of facility claims from Uniform Billing 82 or 92 (UB82 or UB92) form. A Facility Header Record identifier (FACHDID) exists on both this table and the INPATIENT_SERVICES and OUTPATIENT_SERVICES, which can be used to identify the individual service records that each header record comprises. |
| Optum | MEMBER – Contains the enrollment information of each member, including gender, birth year, enrollment start and end dates, plan type.  RX – Contains prescription drug claims.  MEDICAL – Contains both inpatient and outpatient service records. The confinement identifier (CONF_ID) in this table captures inpatient episodes occurring in an acute care hospitalization or skilled nursing facility setting. |
| CCAE = Truven MarketScan Commercial Claims and Encounters  Optum = Optum Clinformatics | |
