# Supplementary material for: The impact of standardizing the definition of visits on the consistency of multi-database observational health research
Source: BMC Med Res Methodol. 2015 Mar 8;15:13. doi: 10.1186/s12874-015-0001-6 (PMC4369827; doi:10.1186/s12874-015-0001-6)
Supplement: Additional file 2: — Codes for Defining Inpatient and Emergency Room Claims Used to Define Standardize Visits. CCAE = Truven MarketScan Commercial Claims and Encounters; Optum = Optum Clinformatics; CPT = Current Procedural Terminology Codes. [file 12874_2015_1_MOESM2_ESM.docx]

**Additional file 2 Codes for Defining Inpatient and Emergency Room Claims Used to Define Standardize Visits**

| **Description** | **Codes Used** |
| --- | --- |
| Inpatient Claims | *Revenue Codes:*  Room & Board (R&B) Charges  0100-0101 – All Inclusive Rate  0110-0169 – R&B – Private, Semiprivate, Ward, Other  0170-0179 – Nursery  0180-0189 – Leave of Absence or Reserved  0190-0219 – Subacute Care, Intensive Care, Coronary Care  Labor Room and Delivery  0720-0729 – Labor Room/Delivery  Inpatient Renal Dialysis  0800-0809 – Inpatient Renal Dialysis |
| Emergency Room Claims | *Revenue Codes:*  0450-0459 – Emergency Room  0981 – Professional Fees – Emergency Room  *Place of Service Codes:*  CCAE  23 – Emergency Room – Hospital  Optum  9-12 – Emergency Room  1226 – Emergency Room  *CPT Codes:*  99281-99285 – Emergency department visit for the evaluation and management of a patient.  *Service Sub-Category Code:*  Any code that ends in a 20 indicates an emergency room visit (CCAE)  10120, 10220, 10320, 10420, 10520 – Facility inpatient ER  20220 – Physician Non-Specialty inpatient ER  12220 – Facility outpatient ER  21220 – Physician Non-Specialty outpatient ER  20120 – Physician Specialty inpatient ER  22320 – Professional outpatient ER  21120 – Physician Specialty outpatient ER  22120 – Professional inpatient ER  30120, 30220, 30520, 30620, 30320, 30420 – Mental Health ER  31120, 31220, 31320, 31420, 31520, 31620 – Substance Abuse ER |
| CCAE = Truven MarketScan Commercial Claims and Encounters  Optum = Optum Clinformatics  CPT = Current Procedural Terminology Codes | |
